# One, Two, Three: Polycomb Proteins Hit All Dimensions of Gene Regulation

**DOI:** 10.3390/genes6030520

**Published:** 2015-07-10

**Authors:** Stefania del Prete, Pawel Mikulski, Daniel Schubert, Valérie Gaudin

**Affiliations:** 1 INRA, UMR1318-AgroParisTech, Institut Jean-Pierre Bourgin (IJPB), INRA-Centre de Versailles-Grignon, Route de St-Cyr, Versailles Cedex F-78026, France; E-Mail: stefania.del-prete@versailles.inra.fr; 2 Institute for Genetics, HHU Duesseldorf, Universitätsstraße 1, Duesseldorf D-40225, Germany; E-Mails: Pawel.Mikulski@uni-duesseldorf.de (P.M.); Dan.Schubert@fu-berlin.de (D.S.)

**Keywords:** Polycomb, chromatin, *Arabidopsis thaliana*, three-dimensional nuclear architecture, Polycomb bodies, topologically associating domain (TAD), chromatin loops, lamins

## Abstract

Polycomb group (PcG) proteins contribute to the formation and maintenance of a specific repressive chromatin state that prevents the expression of genes in a particular space and time. Polycomb repressive complexes (PRCs) consist of several PcG proteins with specific regulatory or catalytic properties. PRCs are recruited to thousands of target genes, and various recruitment factors, including DNA-binding proteins and non-coding RNAs, are involved in the targeting. PcG proteins contribute to a multitude of biological processes by altering chromatin features at different scales. PcG proteins mediate both biochemical modifications of histone tails and biophysical modifications (e.g., chromatin fiber compaction and three-dimensional (3D) chromatin conformation). Here, we review the role of PcG proteins in nuclear architecture, describing their impact on the structure of the chromatin fiber, on chromatin interactions, and on the spatial organization of the genome in nuclei. Although little is known about the role of plant PcG proteins in nuclear organization, much is known in the animal field, and we highlight similarities and differences in the roles of PcG proteins in 3D gene regulation in plants and animals.

## 1. Introduction

In 1947, P. Lewis [1] discovered the Polycomb (*Pc*) gene. While conducting genetic studies in *Drosophila melanogaster*, he found that the *Pc* mutation led to the formation of ectopic sex combs on the second and third legs of adult male flies. Later, while studying the segmentation patterns of Drosophila, it was shown that Pc is a key developmental regulator required to maintain homeotic gene (Hox) repression of the Bithorax complex (BX-C) [2]. Polycomb group (PcG) proteins were unified in a common family on the basis of their similar impact on development and their transcriptional repressive function, but they have various molecular activities [3,4,5,6]. Beyond their originally identified roles in controlling the sequence and timing of developmental switches and in maintaining cell and organ identity both in animals and plants [7,8], it is now clear that PcG proteins accomplish a variety of functions. For instance, recent evidence has implicated plant PcG proteins in abiotic and biotic stress responses [9,10]. During development, stress responses, and other processes, PcG proteins participate in a memory system and establish, maintain, and transmit silent epigenetic chromatin states. However, how PcG repression is established and transmitted *in vivo*, what mechanisms underlie repression, and how PcG activity is reset are only partially understood, even though intensive research efforts in diverse eukaryote models, from flies to plants, have attempted to answer these questions for more than sixty years.

During evolution, the genes encoding PcG proteins diversified from single copy genes to small gene families in animals, plants, and fungi [8]. PcG proteins form large and diverse complexes harboring catalytic subunits, which mediate post-translational modifications of the histone tails, and regulatory subunits, which regulate the transcriptional state of genes. Two main Polycomb repressive complexes (PRCs) each consisting of four core components were initially identified in Drosophila: Polycomb Repressive Complex (PRC) 1 and PRC2. The core components are generally conserved in plants and animals, but some kingdom-specific components evolved to meet the particular needs of the organism’s life cycle (for detailed reviews: [8,11,12,13]). For instance, the plant LIKE HETEROCHROMATIN PROTEIN1 (LHP1) is a functional homolog of Pc, despite its structural homology with the HP1 protein family [14,15]. PcG not only biochemically modifies chromatin [16,17,18], but also induces biophysical changes in chromatin structure [19,20,21,22]. Furthermore, some PcG proteins can be part of other additional and unrelated complexes.

PcG function is antagonized by another group of proteins that modifies chromatin and regulates genes, the trithorax group (TrxG) proteins. The TrxG proteins activate gene expression and, like PcG proteins, are conserved in eukaryotes [23]. PcG and TrxG proteins often have the same target genes, and the activity of these genes is finely tuned by the opposing action of these two protein complexes.

Here, we present a brief overview of the molecular functions, recruitment, and regulation of PcG, with an emphasis on new research that highlights the roles of PcG proteins at different nuclear scales.

## 2. Hierarchy *vs.* Plasticity for More Flexibility in Repression?

### 2.1. Canonical Two-Step Mechanism of Repression

PRC1 and PRC2 often occupy the same target locations and can act in a sequential manner, as proposed in the “canonical model” of PcG repression. The core PRC2 complex trimethylates histone H3 at lysine 27 (H3K27me3) at its target genes in a process that usually takes place on a genomic region called the nucleation site, and the deposited mark spreads over adjacent nucleosomes [24,25,26]. The deposition of H3K27me3 triggers binding of the PRC1 complex, which further ubiquitinates a lysine residue of histone H2A. These modifications result in other complementary factors being recruited, leading to inhibition of transcription and change in chromatin organization. The inhibition mechanism is elusive. On the other hand, TrxG complexes promote transcription, mostly by introducing the H3K4me3 and H3K36me3 activation marks. Remodeling of chromatin renders it more accessible to transcription factors and triggers transcriptional activation; however, this activation does not necessarily rely on the promotion of Polymerase II complex recruitment to target genes, but rather on transcriptional elongation from poised Polymerase II complexes [27,28]. The functions and hierarchical mode of action of PcG and TrxG proteins were extensively reviewed elsewhere [29,30,31,32].

Gene expression dynamics are not only regulated by the antagonistic roles of PcG and TrxG complexes, but also by the active removal of histone modifications deposited by these proteins. For instance, plant histone demethylases (HDMs), including JumonjiC (JmjC) domain containing proteins, counteract the action of PcG proteins in physiological processes, such as the flowering transition, shoot development, cell fate determination, or the circadian clock [33].

### 2.2. Non-Canonical Mechanisms of Repression

For years, the canonical model was considered to describe the only mechanism that regulates the target genes of PcG complexes. Recent studies have uncoupled the functions of PRC1 and PRC2 and revisited the sequential action of the complexes, revealing more complex working mechanisms [17,18,34,35,36,37,38]. Furthermore, PRC1-like complexes, which differ in composition (absence of some of the core subunits present in PRC1 proteins, or the presence of additional subunits), associated activities, or repressive functions, have been reported in mammals, Drosophila, and plants.

In mammals, various additional components can be combined with the core PRC1 subunits to form a large and diverse PRC1 complex family [38,39,40,41]. These additional PRC1 subunits may coordinate other histone modifications and histone crosstalk to repress gene expression. For instance, H3K36 demethylases have been found to associate with PcG proteins in the animal BCOR complex, which regulates a subset of BCL6 target genes [35] or in the Drosophila dRING-associated factor (dRAF) complex [18]. In dRAF, the H3K36 demethylase KDM2 is also required for efficient H2A mono-ubiquitination [18], whereas in BCOR, KDM2B targets the complex to unmethylated CpG islands [42]. The dRAF complex is not the only PRC1-like complex present in Drosophila; others include the Pho-repressive complex (PhoRC) and Polycomb-repressive deubiquitinase complex (PR-DUB) [43,44,45]. Moreover, in mammals, examples of H3K27me3-independent PRC1 targeting or H2A mono-ubiquitination are emerging [17,36,46].

A recent study of the regulation of seed maturation genes in *A. thaliana* revealed that H2Aub and H3K27me3 are deposited on some PcG target genes independently [34]. Indeed, the *clf*/*swn* PRC2 mutant, which has reduced levels of H3K27me3 on several target genes, seems not to be affected in overall levels of H2Aub [34]. Furthermore, plant PRC1 is sometimes recruited before PRC2 [34,47,48,49]. Recent studies have shown that complex relationships exist between PRC1 and PRC2, and that some PRC complexes (*i.e.*, PR-DUB and dRAF) have the capacity to mediate histone modifications other than H3K27 trimethylation and H2A ubiquitination, thus tremendously expanding our understanding of PcG-mediated repression mechanisms.

## 3. Various Ways to Hunt for Targets

PcG targeting has been reported to rely on the presence of *cis*-regulatory elements, *trans*-acting components (DNA-binding proteins, transcription factors, scaffolding proteins, non-coding RNAs (ncRNAs)), or even structural properties of chromatin fiber [50,51,52].

In Drosophila, PRCs are recruited at Polycomb Response Elements (PREs), which are *cis*-regulatory DNA sequences of up to a few hundred base pairs long. PREs contain combinations of several diverse binding motifs for proteins, such as the DNA-binding PcG protein Pho or GAG factor (GAF), which work cooperatively. PREs can be located several tens of kilobases away from the promoter of the target genes, and their properties depend on the sequence context [45,50,53]. The exact combinations of DNA-binding sites and key regulatory elements that determine the recruitment of the PRC complexes remain to be identified.

The DNA-binding PcG protein Pho participates in the targeting of the Drosophila PhoRC complex, and the PhoRC complex may serve as a tethering platform for other PRC complexes at some genomic locations. However, the targeting mechanism for Drosophila PRC1 and PRC2 at PREs and the identity of putative PRE-DNA-binding candidates remain unknown [53]. In mammals, only a few PREs have been identified; however, 97% of PRC2 targets were shown to correspond to annotated CpG islands or similar CG-rich regions. However, the PREs lack a consensus motif [50,54].

In *A. thaliana*, the presence of PREs has not been confirmed. However, GAGA-motifs were recently shown to overlap with the binding sites of FIE, a PRC2 subunit, and these motifs seem to be necessary for H3K27me3 deposition [55]. GAGA-motifs have also been identified in the target genes of LEAFY (LFY), which are repressed by PRC2 [56]. Recently, it was shown that the PRC1-like subunit LHP1 interacts with the GAGA factor BPC6 and that this interaction is essential and sufficient to recruit LHP1 to DNA sequences that contain GAGA-motifs *in vitro* [57]. The repression of *LEAFY COTYLEDON2* (*LEC2*), a key regulator of *A. thaliana* seed development, also requires a negative *cis*-regulatory element, the Repressive LEC2 Element (RLE), which is located 150 bp upstream of the first codon and is associated with CT-rich elements. The RLE triggers H3K27me3 deposition and inhibits transcriptional activity [58]. Conserved Regulatory Elements are also present in the upstream region of the promoters of two *KNOX* genes (*BREVIPEDICELLUS* (*BP*) and *KNOTTED-LIKE FROM ARABIDOPSIS THALIANA2* (*KNAT2*)), which are required for proper organ formation. These sequences are targets of the AS1-AS2 complex, which mediates the repression of *KNOX* genes [59]. Subsequently, the AS1-AS2 complex was shown to interact with multiple core components of PRC2 to establish the repressed chromatin state at *KNOX* genes [60]. This was one of the first demonstrations of a plant PRC2 being recruited by specific DNA-binding proteins. Similarly, VAL1 is needed to recruit BMI1, a PRC1 subunit in *A. thaliana*, and set the repressive state of embryo-specific genes [34]. More evidence that PcG is recruited by transcription factors came from a study of the MADS box protein, AGAMOUS (AG). AG binds two CArG boxes located 1 kb downstream of *WUSCHEL* (*WUS)*, and is required for the regulation of H3K27me3 levels and repression of *WUS* expression [61]. The MADS box transcription factor, SHORT VEGETATIVE PHASE (SVP), and the GRAS transcription factor, SCARECROW (SCR), also physically interact with LHP1 and bind to specific LHP1 target loci, and thus could participate in the recruitment of PRC complexes and in the maintenance of silent chromatin states at their respective target loci [62,63].

Long non-coding RNAs (lncRNAs) have been shown to participate in numerous mechanisms that regulate gene expression, through interactions with various proteins, including histone-modifying molecules [64,65]. It is still a matter of debate whether lncRNAs participate in the recruitment of PcG complexes either in *cis* or in *trans* [66,67]. The first lncRNAs, identified based on their ability to tether PRC2 to target genes, were mammalian Xist and the lncRNA *HOTAIR* [68,69,70]. Since then, numerous studies have examined the interaction between lncRNAs and PRC in mammals, and some have revealed that PRC interacts with several hundred large intergenic ncRNAs [71].

In *A. thaliana*, the list of identified lncRNAs is growing rapidly, with the emergence of *in silico* and RNA-seq technology, but their functions remain poorly understood [72,73,74]. However, two lncRNAs, *COLD ASSISTED INTRONIC NONCODING RNA* (*COLDAIR*) and *COLD INDUCED LONG ANTISENSE INTERGENIC RNA* (*COOLAIR*), are well-characterized regulators of the key floral repressor *FLC* during vernalization [75,76]. *COLDAIR* is a sense-lncRNA transcribed from the vernalization response element (VRE) of the first intron of *FLC*. *COLDAIR* was shown to interact *in vitro* with the CLF PRC2 subunit. Transiently induced by the cold, with peak expression after 20 days of cold exposure, *COLDAIR* was proposed to play a role in recruiting PRC2 to stably silence *FLC* [76]. *COOLAIR* is a set of lncRNA antisense transcripts involved in the early, cold-dependent, and transient transcriptional silencing of *FLC*. Somehow, *COOLAIR* acts as an indirect recruiter of PRC2, but the precise mechanism behind this function is unclear [75,77]. Recently, the lncRNA *APOLO*, which is expressed in response to auxin, was shown to interact with LHP1 [78]. LHP1 also interacts *in vivo* with the RNA-binding protein LIF2, which thus may participate in the formation of a ribonucleoprotein complex involving LHP1. The specificity of the interaction with the RNA partners may thus rely on the three RNA recognition motifs of LIF2 [79]. Recently, specific and promiscuous interactions have been demonstrated in animals, and the strength of the interactions between PcG proteins and lncRNAs was found to depend on the length of the RNA [80]. Whether plant PRC-lncRNA interactions are specific, promiscuous, or non-specific *in vivo* remains to be further investigated. Furthermore, it remains to be determined whether the lncRNA interaction stabilizes PRC complexes with chromatin, participates in the targeting and recognition of the target by a sequence-specific mechanism, or is involved in other scaffolding mechanisms that coordinate different enzymatic activities merits further investigation.

It was recently proposed that PRC2 also recognizes and distinguishes between “open” and “dense” chromatin, with a preference for the latter. Indeed, PRC2 seems to be targeted to specific chromatin features or chromosomal structures [81]. Thus, despite much progress, many open questions remain about how PcG complexes are recruited to silence specific genes.

## 4. On the Path from 1D to 3D

### 4.1. Never Walk Alone—Polycomb and Chromatin Domains

Initial discoveries of PcG targets at the gene-scale were soon followed by a genome-wide analysis of H3K27me3 occupancy and binding of several PcG-proteins [15,54,82,83]. As expected, a substantial overlap between PRC2-bound fragments and H3K27me3 profiles was found. A correlation between PRC1-binding and H3K27me3 occupancy was also reported, with some exceptions, suggesting that PRC1 has other functions.

**Figure 1:**
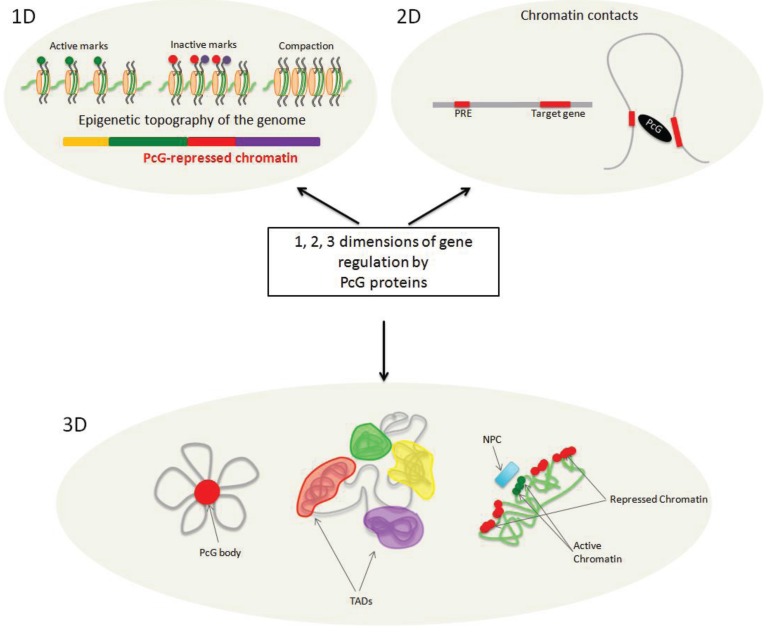
Polycomb Group (PcG) proteins affect chromatin regulation in three dimensions. PcG proteins participate in the establishment of the epigenetic topography of the genome by depositing biochemical modifications on histones and inducing chromatin compaction (**1D**). The presence of PcG-associated marks defines a specific chromatin state, called Polycomb-repressed chromatin. PcG proteins can mediate chromatin looping (**2D**). PcG complexes are recruited to *cis*-regulatory elements by various mechanisms (e.g., those involving Polycomb Response Elements (PREs), transcription factors, lncRNAs) to regulate their target genes and change the conformation of the chromatin fiber. PcG proteins act in the nuclear space (**3D**). They can aggregate with their targets to form Polycomb bodies. Chromatin is organized into distinct topologically associated domains (TADs), some of which are Polycomb-repressed chromatin domains. Spatial localization of chromatin at the nuclear periphery is correlated with repressive histone marks and, in the vicinity of the nuclear pore complexes (NPC), with active histone marks.

Moreover, it was shown that, in plants, H3K27me3 is deposited predominantly on euchromatin, spans whole genes, and targets up to 20%–30% of all genes in *A. thaliana* [84,85,86,87] and 10% in human [82,88], stressing the importance of the Polycomb pathway in genome regulation. The H3K27me3 occupancy pattern was also compared to the profiles of various histone marks, genomic features of underlying sequences, protein binding distributions, and transcriptional activity of target genes to identify common patterns and predict gene expression status, resulting in the identification of so-called chromatin states or domains that include regions of similar characteristics. In the first such study in Drosophila, a Polycomb-repressed chromatin state was identified, in which H3K27me3-targeted genomic fragments were associated with genic regions, lower transcription, occupancy of other repressive marks, and binding regions of PcG proteins (e.g., H3K27me2, Pc, E(Z), PCL [89]), and inversely associated with the active marks H3K4me1/2/3, H3K36me3, and H3K27Ac [90]). Several studies distinguished bivalent domains, in which repressive marks are accompanied by active ones, which are inactive or poised for transcription [91]. One of the main differences between the studied species was that animal H3K27me3-occupied regions consisted of large blocks formed by adjacent regions of a similar epigenetic landscape, whereas the *A. thaliana* (epi-)genome seemed to be organized into small domains of different states interspersed with each other (apart from constitutive heterochromatin, which was spread over the pericentromeric region of the chromosome) [92]. Therefore, the epigenetic topography of the genomes has emerged and the development of high throughput sequencing has tremendously refined it (Figure 1).

### 4.2. PcG and Chromatin Fiber Packaging

Chromatin compaction and formation of higher-order chromatin structures are proposed to participate in the repression by PcG, by reducing or interfering with DNA accessibility to the transcription machinery. Indeed, *in vitro* studies have shown that the Drosophila PRC1 complex and, more specifically, the Posterior sex combs (PSC) subunit, have the ability to compact nucleosomal arrays and inhibit the chromatin remodeling mediated by the SWI/SNF complex [93]. The intrinsically disordered *C*-terminal region of PSC changes the beads-on-a-string chromatin conformation into high-order chromatin structures [21]. Linker DNA and histone tails might also participate in chromatin compaction; however, Drosophila PRC1 directly interacts with nucleosomes and its activity seems to be independent of histone tail modifications [21]. The important role of PRC1 in the compaction of nucleosomal arrays is conserved across metazoans and plants [19,21,93,94], but is carried out by different PRC1 subunits, such as M33, a Polycomb homolog in mouse, or the PRC1 component EMBRYONIC FLOWER1 (EMF1) in *A. thaliana* [19,94]. Detailed studies of PSC and M33 revealed that one of the main characteristics of a “chromatin compactor” protein is the presence of highly positively charged domains [94]. Using the FISH technique, one study revealed that the PRC1 Ring1B subunit is also involved in *in vivo* compaction and regulation of Hox loci in murine embryonic stem cells (ESCs). This compaction activity is independent of Ring1B histone H2Aub catalytic activity [95]. Therefore, increasing evidence suggests that PRC1 participates in higher-order chromatin structure organization and that PRC1 has packaging effects on chromatin, which may be sufficient to mediate repression, at least at some specific target genes.

In support of a role of PcG in chromatin compaction, genome-wide studies demonstrated that H3K27me3-marked chromatin regions have low DNA accessibility both in Drosophila and *A. thaliana* [96,97]. In addition, chromatin in *lhp1* mutants is more sensitive to microccocal nuclease digestion than is wild-type chromatin, suggesting a possible role for LHP1 in chromatin compaction. Although LHP1 is functionally similar to Pc, it is classified as a HP1 protein, based on its structure [14,15]. The chromo shadow domain (CSD) of the HP1 protein family is involved in homo-dimerization, whereas the chromo domain (CD) recognizes methylated lysine residues on histone tails. Interestingly, the binding of two CDs of Swi6, the *Schizosaccharomyces pombe* HP1 homolog, to nearby modified histone H3 nucleosomes creates a protein interface for tetramerization of two Swi6 homo-dimers connected by their CSDs [98]. The coordinated action of CD and CSD can thus lead to heterochromatin spreading throughout a stepwise oligomerization process from an auto-inhibited homodimer to chromatin-associated oligomers, which is somewhat analogous to the self-association process of tubulin dimers [99]. Whether this model, which is still controversial [100], is conserved for the HP1 family and plant LHP1 remains to be established.

Interestingly, it was recently shown that PRC2 has a higher activity on dense oligonucleosomes than on dispersed oligonucleosomes, suggesting that PRC2 can sense the chromatin environment and that its allosteric activation depends on the density of the substrate nucleosomes [81]. This study also revealed that local chromatin compaction precedes the establishment of H3K27me3 and provides a better substrate for PRC2 activity [81]. Therefore, PRC1 may initiate local chromatin compaction, which could then lead to the establishment and propagation of the H3K27me3 mark. This would support recent data suggesting that PRC1 acts before PRC2 [34,47,48,101]. However, the structural role of PRC complexes, at the local chromatin scale or at a higher order of chromatin organization, requires further investigation.

### 4.3. It Takes Two to Tango—Chromatin Loops Mediated by PcG Proteins

Besides their roles in linear chromatin topography, increasing evidence suggests that PcG also affects the three-dimensional (3D) organization of chromatin at the nuclear level. Although the spatial genome regulation mediated by PcG proteins remains poorly understood in plants, we highlight recent findings, while taking existing evidence in other species into account.

PcG proteins can organize chromatin into 3D long-range loops, both in plants and in animals. These loops may also participate in PcG-mediated repression mechanisms (reviewed in [102]). Two techniques are used to investigate 3D chromatin organization, *i.e.*, fluorescence *in situ* hybridization (FISH), which allows direct visualization of the spatial organization of genomic sequences by cytology and microscopy approaches in individual nuclei, and methods derived from chromosome conformation capture (3C), which allow high-throughput and high-resolution analyses of genomic interactions at local to genome-wide scales [103,104,105].

Using both methods, Lanzuolo and colleagues [106] provided one of the first direct lines of evidence of loop formation in Drosophila. All major regulatory PcG-bound DNA elements at the Bithorax (BX-C) locus were found to physically interact with each other via chromatin long-range interactions. After artificial transcriptional reactivation of the BX-C locus, different conformations of the locus were observed, with the active PREs and promoters losing contact with each other [106]. This early study directly linked transcriptional regulation with the regulation of the chromatin loop formation involving PREs. A direct role for PcG in loop formation was provided by the study of the regulation of the human *GATA*-4 locus, a target of the PRC2 EZH2 subunit [107]. When EZH2 levels are depleted, the long-range genomic contacts at the *GATA*-4 locus are disrupted, leading to transcriptional reactivation [107]. PRC2 has subsequently been shown to change chromatin conformation at the murine *HoxD* locus [108]. Analysis of a PRC2 mutant by Carbon-Copy 3C (5C) (which allows for interaction studies between many selected loci of a specific region) and by FISH showed a strong reduction of the 5C interactions over the *HoxD* region [108]. The loops involve physical contacts between PRE and PcG target genes, but can also form between genes and regulatory elements such as enhancers, silencers, and chromatin insulators [109,110,111].

In plants, the presence of loops directly mediated by PcG proteins has not yet been demonstrated. However, in *Zea mays* (maize) and *A. thaliana*, examples of loops between flanking regions of genes have been identified. In maize, husk-specific physical interaction between an enhancer located 100 kb upstream of the transcription start site (TSS) and the TSS is required for the high expression of the *b1* gene [112]. In *A. thaliana*, a gene loop has been identified that involves the physical interaction of the 5' and 3' flanking regions of the *FLC* locus [111,113]. Loop formation is regulated by vernalization, as it is disrupted within the first 2 weeks of cold exposure. The disruption occurs in parallel with the switch from an expressed to a Polycomb-silenced state of the *FLC* locus. Concurrently, clustering of the repressed *FLC* alleles to a single nuclear location occurs, and this is disrupted in the *vrn2* and *vrn5* mutants [114]. This finding suggests that the VRN2 and VRN5 PRC2 components influence the nuclear organization of repressed *FLC* alleles. In addition, the PcG protein LHP1 was shown to facilitate auxin-regulated loop formation between a long non-coding RNA and a gene involved in the root development pathway [78]. The presence of a loop was correlated with lower expression of these loci, increased DNA methylation, and higher levels of H3K27me3, suggesting that several repressive pathways, including the one mediated by PRC2, are involved in the process.

Understanding the formation of these chromatin hubs is an exciting and active area of research, due to their potential regulatory function and impact on the structure and evolution of genomes. However, it is still unclear whether PcG proteins are required for the formation or stabilization of the loops, and whether the 3D chromatin conformational changes are causes or consequences of altered gene expression. Furthermore, the methods used to examine chromatin loops are technically challenging. Discrepancies have been observed in the frequency of genomic interactions [115,116,117]. In addition, a limited number of studies compared the data resulting from FISH and 3C-derived experiments. These methods give concordant data in some cases, but also yield divergent results [108]. Therefore, in future studies of chromatin looping formation and dynamics, both techniques should be used and the results should be compared [108].

## 5. In the Third Dimension: Polycomb Mediates Higher Order Chromatin Organization

### 5.1. Polycomb and Topologically Associating Domains

Derivatives of 3C techniques are used to establish genome-wide maps of chromatin contacts or physical interactions within the genome. In Drosophila, mouse, and human, these studies highlighted that the genome is organized into topologically associating domains (TADs) that participate in its functional architecture in the nuclear space [115,118,119,120,121,122,123,124]. TADs correspond to linear chromatin regions that fold as specific 3D structures that mainly favor internal interactions within a particular TAD. The mean size of TADs varies among species, ranging from 60 kb in Drosophila to 900 kb in mouse and human.

The distribution of TADs along the chromosome is associated with specific underlying histone modifications, density of nucleosomes, and DNA modification: Each TAD thus corresponds to one of the four major epigenetic topographical chromatin types: Active chromatin, Polycomb-repressed chromatin, null chromatin, or heterochromatin [122,125]. In *D. melanogaster* embryonic nuclei, Polycomb-repressed domains are highly correlated with specific TADs. Furthermore, it was proposed that the intrinsic folding regime of inactive TADs is specific and different from that of active TADS, with the probability of contacts forming between inactive TADs being lower and with a stronger association with the chromosome territory [120]. The situation is more complex in mammals, due to the discrepancy between the size of TADs (ranging from 100 kb to 10 Mb) and H3K27me3 domains (1 kb to 100 kb). Only a few overlaps have been reported between the two entities [119,126].

Sharp boundaries containing insulator sites and housekeeping genes have been shown to mark the limits between TADs [120,124]. Moreover, the modularity of TAD organization is conserved in different cell types in animals, but intra-TAD interactions can vary greatly according to cell type [118,119].

Until now, only a few conformation capture studies have been performed in plants [127,128,129]. In *A. thaliana*, the presence of TADs has not yet been clearly established, the results still dependent on the resolution of the Hi-C methods used [127,128]. At low resolution and at long-range distances (>100 kb), it seems that the *A. thaliana* genome contains relatively small interacting regions, which are distributed over multiple sites in the genome. The main contact regions depend mostly on the presence of DNA methylation and of H3K9me2 or H3K27me1 marks, which allows for the identification of interactive heterochromatin islands (IHI). These main interactions occur between telomeres, whereas pericentromeric heterochromatin regions interact weakly with the rest of the genome, but strongly with each other [127]. AtMORC6, a conserved Microrchidia adenosine triphosphatase required for heterochromatin condensation, seems to play a key role in establishing these heterochromatic genome contacts [130]. A limited number of H3K27me3 regions showed interactions, and these were mostly scattered throughout the genome and were dependent on PRC2 activity [127]. However, using the Hi-C method at higher resolution, about half of the contact regions were shown to be enriched in H3K27me3, H3.1, and H3.3 [128]. Although TADs were not found to be a prevailing structural feature of the *A. thaliana* genome, hundreds of insulator-like regions or regions analogous to TAD boundaries have been discovered [128]. These regions are enriched for accessible chromatin sites, various activating epigenetic marks, and highly expressed genes, whereas TAD-like fragments are characterized by opposite patterns, indicating a repressed chromatin state.

Overall, it remains challenging to identify TADs and specific Polycomb-associated TADs in plants. Conformation capture experiments in plants with larger genomes and at higher resolution in *A. thaliana* may cast light on TAD organization.

### 5.2. Polycomb Clustering

The 3D organization of PcG target regions has been examined by imaging the subnuclear localization of Polycomb proteins. Interestingly, some Polycomb complex components tend to aggregate in foci instead of being randomly dispersed over the nuclear space [131,132,133,134]. These spots of increased signal were called PcG bodies. They differ in size and number in different cell types; as a general rule, fewer and larger foci are present in undifferentiated cells, whereas more numerous and smaller foci occur in differentiated cells [105,132].

Subsequent experiments showed that PcG foci serve as structures in which different Polycomb targets are clustered and co-repressed. A prime example comes from the Drosophila Antp and Abd-B loci. These PcG targets, despite being located 10 Mb apart from each other, co-localize to foci in embryo heads, where they are inactive, and dissociate in differentiated tissue, where at least one of them becomes activated. Further evidence emerged from chromatin conformation capture studies. As mentioned above, it was shown that PREs of different PcG targets can interact with each other even over long distances [115,123], confirming the imaging data. Recently, more than hundred proteins have been identified as regulators of the 3D distribution of PcG proteins in Drosophila [135]. In particular, proteins involved in the sumoylation pathway were shown to be critical for the Pc chromatin binding affinity, residence time, and 3D nuclear distribution. Indeed, Pc foci form enlarged aggregates in the absence of SUMO, whereas they are more dispersed when the activity of the SUMO peptidase Velo is reduced [135]. Interestingly, it was also demonstrated that the Polyhomeotic (Ph) protein assembly at PREs is defective in the absence of its *O*-GlcNAcylation and that its post-translational modification is required for the ordered and functional assembly of Ph via its SAM domain [136].

In plants, the existence of Polycomb bodies remains elusive. However, several lines of evidence suggest that Polycomb components or their targets undergo clustering. Rosa *et al.* [114] showed clustering of the PcG target, *FLC*, that was induced by vernalization and quantitatively correlated with the length of cold exposure. Higher clustering frequency was tightly associated with increased accumulation of H3K27me3 on the *FLC* nucleation site and clustering was impaired in the PcG mutants *vrn2* and *vrn5*, but not *lhp1*. In contrast, imaging of LHP1-GFP in *A. thaliana* showed that LHP1 nuclear distribution patterns vary from a rather uniform pattern in meristematic cells to patterns with multiple distinguishable foci in differentiated cells [137]. The relationship between LHP1 distribution and the differentiation status of the cell is reminiscent of the link between PcG bodies and cell differentiation in animals.

We believe that further development of imaging and 3D interaction techniques will elucidate the clustering of Polycomb targets in plants and the underlying mechanisms. However, genome arrangement in different species might also be the result of different evolutionary paths, which might lead to distinct mechanisms of repressive 3D interactions.

### 5.3. Polycomb Regulation and Spatial Distribution

Another peculiar aspect of Polycomb regulation involves the distribution of PcG targets in the nuclear space, whose spatial rules have not yet clearly been established. However, the nuclear periphery and nuclear lamina (NL) have been shown to play important roles in gene regulation.

The NL is a protein mesh residing inside the nuclear membrane. In animals, the NL is composed of proteins called lamins, which localize to thin ring structures that cover the inner surface of the nuclear membrane. Despite a lack of substantial sequence homology to animal counterparts, a couple of proteins in plants are believed to fulfill the role of lamins, because of their lamin-like localization and the altered nuclear morphology and size in the corresponding mutants. Prominent examples of plant lamin-like proteins include NMCP1 (*D. carota*) and CROWDED NUCLEI1-4 (CRWN1-4) (*A. thaliana*). The NL is not a uniform structure; it spans the nuclear membrane and is interconnected with Nuclear Pore Complexes (NPCs), which are protein structures that form channels for the exchange of molecules between the nucleoplasm and cytoplasm (for reviews, [138,139,140]).

Interestingly, a physical association between the DNA and the nuclear periphery was shown to influence gene regulation. State-of-the-art examples come from studies on human [141] and Drosophila [142], in which the authors mapped the interactions between the genome and lamins using the DamID technique [143,144] and subsequent genome-wide profiling. Parts of the genome bound by lamins show lower expression and an abundance of repressive chromatin marks, including H3K27me3 [141,145,146,147]. Consistently, depletion of Drosophila [148] and *C. elegans* [149] lamin orthologues caused up-regulation of genes at the nuclear periphery and artificial tethering of transgenes to the periphery resulted in decreased expression [150,151,152,153].

Localization to the nuclear periphery was also associated with gene activation in several studies. For instance, several groups showed that the inducible yeast genes *INO1* and *GAL1* are recruited to the vicinity of NPCs upon gene induction and remain there for subsequent reactivation [154,155,156,157]. Moreover, global chromatin organization seems also to be tissue specific—in rod cells of nocturnal animals, there is an inverted chromatin arrangement, so that repression is correlated with the nuclear interior and activation with the periphery [158]. Rod cells lack lamins and are responsible for harvesting light; the inverted chromatin arrangement is believed to make rods more efficient. Only a few studies identified a connection between the Polycomb repression mechanism and the nuclear periphery [159,160].

In plants, these studies are still in their infancy. One example of gene repositioning related to gene expression has been reported in *A. thaliana* leaf mesophyll cells [161]. Light-inducible loci moved from the nuclear interior to the periphery upon transcriptional activation [161]. Whether these spatial movements are correlated with chromatin modifications remains to be elucidated. Interestingly, the E3 ubiquitin ligase HIGH EXPRESSION OF OSMOTICALLY RESPONSIVE GENES 1 (HOS1) interacts with components of the *A. thaliana* NPC, thereby facilitating mRNA export. HOS1 activates *FLC* expression by remodeling chromatin at the *FLC* locus during short-term cold stress. The activation takes place by antagonizing the silencing role of FVE, a PcG-related protein and the displacement of its partner, the histone deacetylase HDA6. This, in turn, leads to increased acetylation of histone H3 on *FLC*, which results in higher expression [162]. Whether the two functions in nuclear export and chromatin remodeling are linked is an open question. Finally, *A. thaliana* structural components, such as the CRWN1 and CRWN4 proteins, which control the size of the nucleus, localized at the nuclear periphery and seemed to influence chromosomal organization [129]. Indeed, Hi-C experiments revealed that the *crwn4* and *crwn1* mutants exhibited increased *trans*-chromosomal interaction frequencies, suggesting higher levels of chromosomal compaction [129]. Thus, no link with plant PcG proteins has yet been demonstrated.

## 6. Conclusions

We can view the Polycomb-mediated impact on chromatin dynamics and genome regulation as an integration of three layers or dimensions of repression (Figure 1). The first one is based on the information stored on the linear genome and epigenome. Important aspects of this first dimension include recruitment at specific genomic regions, modification of histone tails, compaction of nucleosomes, and interplay with other factors to form Polycomb-repressed chromatin domains that shape the epigenetic topography of the genome. The second dimension involves the formation of loops on a locus scale, when Polycomb *cis*-regulatory elements interact with each other. Lastly, the third dimension relies on the organization of Polycomb-based TADs and the spatial localization of Polycomb-repressed domains in specific compartments of the nuclear space.

With the development of new methodology and genome-wide approaches combined with modeling to study the 3D arrangement of proteins and genomes, it has become possible to investigate the third dimension of Polycomb repression, and this has deepened our understanding of Polycomb action. PcG proteins continuously surprise us on all layers of repression, by deviating from dogmas and showing non-canonical structures, hierarchies, or mechanisms. As the methodology is now accessible for plants as well, we look forward to the challenge of deciphering the mechanism underlying the 3D PcG-mediated repression in plants, which will certainly bring novel surprises.

## References

[1] Lewis E.B. (1947). New mutants: Reports of P. lewis. Drosoph. Inf. Serv..

[2] Lewis E.B. (1978). A gene complex controlling segmentation in *Drosophila*. Nature.

[3] Schwartz Y.B., Pirrotta V. (2007). Polycomb silencing mechanisms and the management of genomic programmes. Nat. Rev. Genet..

[4] Köhler C., Villar C.B. (2008). Programming of gene expression by Polycomb group proteins. Trends Cell Biol..

[5] Schwartz Y.B., Pirrotta V. (2013). A new world of Polycombs: Unexpected partnerships and emerging functions. Nat. Rev. Genet..

[6] Pu L., Sung Z.R. (2015). PcG and trxG in plants—Friends or foes. Trends Genet..

[7] Xiao J., Wagner D. (2015). Polycomb repression in the regulation of growth and development in *Arabidopsis*. Curr. Opin. Plant Biol..

[8] Hennig L., Derkacheva M. (2009). Diversity of Polycomb group complexes in plants: Same rules, different players?. Trends Genet..

[9] De Lucia F., Gaudin V., Palmiro Poltronieri N.B., Corrado F. (2013). Epigenetic control by plant Polycomb proteins: New perspectives and emerging roles in stress response. From Plant Genomics to Plant Biotechnology.

[10] Kleinmanns J.A., Schubert D. (2014). Polycomb and Trithorax group protein-mediated control of stress responses in plants. Biol. Chem..

[11] Molitor A., Shen W.H. (2013). The polycomb complex PRC1: Composition and function in plants. J. Genet. Genomics.

[12] Bemer M., Grossniklaus U. (2012). Dynamic regulation of Polycomb group activity during plant development. Curr. Opin. Plant Biol..

[13] Pien S., Grossniklaus U. (2007). Polycomb group and trithorax group proteins in *Arabidopsis*. Biochim. Biophys. Acta.

[14] Gaudin V., Libault M., Pouteau S., Juul T., Zhao G., Lefebvre D., Grandjean O. (2001). Mutations in LIKE HETEROCHROMATIN PROTEIN 1 affect flowering time and plant architecture in *Arabidopsis*. Development.

[15] Zhang X., Germann S., Blus B.J., Khorasanizadeh S., Gaudin V., Jacobsen S.E. (2007). The *Arabidopsis* LHP1 protein colocalizes with histone H3 Lys27 trimethylation. Nat. Struct. Mol. Biol..

[16] Bratzel F., Lopez-Torrejon G., Koch M., del Pozo J.C., Calonje M. (2010). Keeping cell identity in *Arabidopsis* requires PRC1 RING-finger homologs that catalyze H2A monoubiquitination. Curr. Biol..

[17] Tavares L., Dimitrova E., Oxley D., Webster J., Poot R., Demmers J., Bezstarosti K., Taylor S., Ura H., Koide H. (2012). RYBP-PRC1 complexes mediate H2A ubiquitylation at polycomb target sites independently of PRC2 and H3K27me3. Cell.

[18] Lagarou A., Mohd-Sarip A., Moshkin Y.M., Chalkley G.E., Bezstarosti K., Demmers J.A., Verrijzer C.P. (2008). dKDM2 couples histone H2A ubiquitylation to histone H3 demethylation during Polycomb group silencing. Genes Dev..

[19] Beh L.Y., Colwell L.J., Francis N.J. (2012). A core subunit of Polycomb repressive complex 1 is broadly conserved in function but not primary sequence. Proc. Natl. Acad. Sci. USA.

[20] Bantignies F., Cavalli G. (2011). Polycomb group proteins: Repression in 3D. Trends Genet..

[21] Francis N.J., Kingston R.E., Woodcock C.L. (2004). Chromatin compaction by a polycomb group protein complex. Science.

[22] Schuettengruber B., Cavalli G. (2013). Polycomb domain formation depends on short and long distance regulatory cues. PLoS ONE.

[23] Ringrose L., Paro R. (2004). Epigenetic regulation of cellular memory by the Polycomb and Trithorax group proteins. Ann. Rev. Genet..

[24] Finnegan E.J., Dennis E.S. (2007). Vernalization-Induced Trimethylation of Histone H3 Lysine 27 at FLC Is Not Maintained in Mitotically Quiescent Cells. Curr. Biol..

[25] De Lucia F., Crevillen P., Jones A.M., Greb T., Dean C. (2008). A PHD-Polycomb Repressive Complex 2 triggers the epigenetic silencing of FLC during vernalization. Proc. Natl. Acad. Sci. USA.

[26] Pinter S.F., Sadreyev R.I., Yildirim E., Jeon Y., Ohsumi T.K., Borowsky M., Lee J.T. (2012). Spreading of X chromosome inactivation via a hierarchy of defined Polycomb stations. Genome Res..

[27] Dorighi K.M., Tamkun J.W. (2013). The trithorax group proteins Kismet and ASH1 promote H3K36 dimethylation to counteract Polycomb group repression in *Drosophila*. Development.

[28] Srinivasan S., Dorighi K.M., Tamkun J.W. (2008). *Drosophila* Kismet regulates histone H3 lysine 27 methylation and early elongation by RNA polymerase II. PLoS Genet..

[29] Mozgova I., Kohler C., Hennig L. (2015). Keeping the gate closed: Functions of the Polycomb repressive complex PRC2 in development. Plant J..

[30] Khanna N., Hu Y., Belmont A.S. (2014). HSP70 transgene directed motion to nuclear speckles facilitates heat shock activation. Curr. Biol..

[31] Schuettengruber B., Martinez A.M., Iovino N., Cavalli G. (2011). Trithorax group proteins: Switching genes on and keeping them active. Nat. Rev. Mol. Cell Biol..

[32] Khan A.A., Lee A.J., Roh T.Y. (2015). Polycomb group protein-mediated histone modifications during cell differentiation. Epigenomics.

[33] Chen X., Hu Y., Zhou D.X. (2011). Epigenetic gene regulation by plant Jumonji group of histone demethylase. Biochim. Biophys. Acta.

[34] Yang C., Bratzel F., Hohmann N., Koch M., Turck F., Calonje M. (2013). VAL- and AtBMI1-mediated H2Aub initiate the switch from embryonic to postgerminative growth in *Arabidopsis*. Curr. Biol..

[35] Gearhart M.D., Corcoran C.M., Wamstad J.A., Bardwell V.J. (2006). Polycomb group and SCF ubiquitin ligases are found in a novel BCOR complex that is recruited to BCL6 targets. Mol. Cell. Biol..

[36] Gao Z., Zhang J., Bonasio R., Strino F., Sawai A., Parisi F., Kluger Y., Reinberg D. (2012). PCGF homologs, CBX proteins, and RYBP define functionally distinct PRC1 family complexes. Mol. Cell.

[37] Calonje M., Sanchez R., Chen L., Sung Z.R. (2008). EMBRYONIC FLOWER1 participates in polycomb group-mediated AG gene silencing in *Arabidopsis*. Plant Cell.

[38] Ogawa H., Ishiguro K., Gaubatz S., Livingston D.M., Nakatani Y. (2002). A complex with chromatin modifiers that occupies E2F- and Myc-responsive genes in G0 cells. Science.

[39] Gil J., O’Loghlen A. (2014). PRC1 complex diversity: Where is it taking us?. Trends Cell Biol..

[40] Levine S.S., Weiss A., Erdjument-Bromage H., Shao Z., Tempst P., Kingston R.E. (2002). The core of the polycomb repressive complex is compositionally and functionally conserved in flies and humans. Mol. Cell. Biol..

[41] Wang H., Wang L., Erdjument-Bromage H., Vidal M., Tempst P., Jones R.S., Zhang Y. (2004). Role of histone H2A ubiquitination in Polycomb silencing. Nature.

[42] Farcas A.M., Blackledge N.P., Sudbery I., Long H.K., McGouran J.F., Rose N.R., Lee S., Sims D., Cerase A., Sheahan T.W. (2012). KDM2B links the Polycomb Repressive Complex 1 (PRC1) to recognition of CpG islands. eLife.

[43] Scheuermann J.C., Gutierrez L., Muller J. (2012). Histone H2A monoubiquitination and Polycomb repression: The missing pieces of the puzzle. Fly.

[44] Klymenko T., Papp B., Fischle W., Kocher T., Schelder M., Fritsch C., Wild B., Wilm M., Muller J. (2006). A Polycomb group protein complex with sequence-specific DNA-binding and selective methyl-lysine-binding activities. Genes Dev..

[45] Schwartz Y.B., Pirrotta V. (2008). Polycomb complexes and epigenetic states. Curr. Opin. Cell Biol..

[46] Schoeftner S., Sengupta A.K., Kubicek S., Mechtler K., Spahn L., Koseki H., Jenuwein T., Wutz A. (2006). Recruitment of PRC1 function at the initiation of X inactivation independent of PRC2 and silencing. EMBO J..

[47] Kalb R., Latwiel S., Baymaz H.I., Jansen P.W., Muller C.W., Vermeulen M., Muller J. (2014). Histone H2A monoubiquitination promotes histone H3 methylation in Polycomb repression. Nat. Struct. Mol. Biol..

[48] Cooper S., Dienstbier M., Hassan R., Schermelleh L., Sharif J., Blackledge N.P., de Marco V., Elderkin S., Koseki H., Klose R. (2014). Targeting polycomb to pericentric heterochromatin in embryonic stem cells reveals a role for H2AK119u1 in PRC2 recruitment. Cell Rep..

[49] Blackledge N.P., Farcas A.M., Kondo T., King H.W., McGouran J.F., Hanssen L.L., Ito S., Cooper S., Kondo K., Koseki Y. (2014). Variant PRC1 complex-dependent H2A ubiquitylation drives PRC2 recruitment and polycomb domain formation. Cell.

[50] Simon J.A., Kingston R.E. (2009). Mechanisms of polycomb gene silencing: Knowns and unknowns. Nat. Rev. Mol. Cell Biol..

[51] Kim D.H., Sung S. (2014). Polycomb-mediated gene silencing in *Arabidopsis thaliana*. Mol. Cells.

[52] He C., Huang H., Xu L. (2013). Mechanisms guiding Polycomb activities during gene silencing in *Arabidopsis thaliana*. Front. Plant Sci..

[53] Kassis J.A., Brown J.L. (2013). Polycomb group response elements in *Drosophila* and vertebrates. Adv. Genet..

[54] Ku M., Koche R.P., Rheinbay E., Mendenhall E.M., Endoh M., Mikkelsen T.S., Presser A., Nusbaum C., Xie X., Chi A.S. (2008). Genomewide analysis of PRC1 and PRC2 occupancy identifies two classes of bivalent domains. PLoS Genet..

[55] Deng W., Buzas D.M., Ying H., Robertson M., Taylor J., Peacock W.J., Dennis E.S., Helliwell C. (2013). *Arabidopsis* Polycomb Repressive Complex 2 binding sites contain putative GAGA factor binding motifs within coding regions of genes. BMC Genomics.

[56] Winter C.M., Austin R.S., Blanvillain-Baufume S., Reback M.A., Monniaux M., Wu M.F., Sang Y., Yamaguchi A., Yamaguchi N., Parker J.E. (2011). LEAFY target genes reveal floral regulatory logic, cis motifs, and a link to biotic stimulus response. Dev. Cell.

[57] Hecker A., Brand L.H., Peter S., Simoncello N., Kilian J., Harter K., Gaudin V., Wanke D. (2015). The *Arabidopsis* GAGA-binding factor BPC6 recruits PRC1 component LHP1 to GAGA DNA-motifs. Plant Physiol..

[58] Berger N., Dubreucq B., Roudier F., Dubos C., Lepiniec L. (2011). Transcriptional regulation of *Arabidopsis* LEAFY COTYLEDON2 involves RLE, a cis-element that regulates trimethylation of histone H3 at lysine-27. Plant Cell.

[59] Guo M., Thomas J., Collins G., Timmermans M.C. (2008). Direct repression of KNOX loci by the ASYMMETRIC LEAVES1 complex of *Arabidopsis*. Plant Cell.

[60] Hyun Y., Yun H., Park K., Ohr H., Lee O., Kim D.H., Sung S., Choi Y. (2013). The catalytic subunit of *Arabidopsis* DNA polymerase alpha ensures stable maintenance of histone modification. Development.

[61] Liu X., Kim Y.J., Muller R., Yumul R.E., Liu C., Pan Y., Cao X., Goodrich J., Chen X. (2011). AGAMOUS Terminates Floral Stem Cell Maintenance in *Arabidopsis* by Directly Repressing WUSCHEL through Recruitment of Polycomb Group Proteins. Plant Cell.

[62] Liu C., Xi W., Shen L., Tan C., Yu H. (2009). Regulation of floral patterning by flowering time genes. Dev. Cell.

[63] Cui H., Benfey P.N. (2009). Interplay between SCARECROW, GA and LIKE HETEROCHROMATIN PROTEIN 1 in ground tissue patterning in the *Arabidopsis* root. Plant J..

[64] Peschansky V.J., Wahlestedt C. (2014). Non-coding RNAs as direct and indirect modulators of epigenetic regulation. Epigenetics.

[65] Marchese F.P., Huarte M. (2014). Long non-coding RNAs and chromatin modifiers: Their place in the epigenetic code. Epigenetics.

[66] Brockdorff N. (2013). Noncoding RNA and Polycomb recruitment. RNA.

[67] Nazer E., Lei E.P. (2014). Modulation of chromatin modifying complexes by noncoding RNAs in trans. Curr. Opin. Genet. Dev..

[68] Rinn J.L., Kertesz M., Wang J.K., Squazzo S.L., Xu X., Brugmann S.A., Goodnough L.H., Helms J.A., Farnham P.J., Segal E. (2007). Functional demarcation of active and silent chromatin domains in human HOX loci by noncoding RNAs. Cell.

[69] Chu C., Qu K., Zhong F.L., Artandi S.E., Chang H.Y. (2011). Genomic maps of long noncoding RNA occupancy reveal principles of RNA-chromatin interactions. Mol. Cell.

[70] Tsai M.C., Manor O., Wan Y., Mosammaparast N., Wang J.K., Lan F., Shi Y., Segal E., Chang H.Y. (2010). Long noncoding RNA as modular scaffold of histone modification complexes. Science.

[71] Khalil A.M., Guttman M., Huarte M., Garber M., Raj A., Rivea Morales D., Thomas K., Presser A., Bernstein B.E., van Oudenaarden A. (2009). Many human large intergenic noncoding RNAs associate with chromatin-modifying complexes and affect gene expression. Proc. Natl. Acad. Sci. USA.

[72] Liu J., Jung C., Xu J., Wang H., Deng S., Bernad L., Arenas-Huertero C., Chua N.H. (2012). Genome-wide analysis uncovers regulation of long intergenic noncoding RNAs in *Arabidopsis*. Plant Cell.

[73] Wang Y., Fan X., Lin F., He G., Terzaghi W., Zhu D., Deng X.W. (2014). *Arabidopsis* noncoding RNA mediates control of photomorphogenesis by red light. Proc. Natl. Acad. Sci. USA.

[74] Di C., Yuan J., Wu Y., Li J., Lin H., Hu L., Zhang T., Qi Y., Gerstein M.B., Guo Y. (2014). Characterization of stress-responsive lncRNAs in *Arabidopsis thaliana* by integrating expression, epigenetic and structural features. Plant J..

[75] Swiezewski S., Liu F., Magusin A., Dean C. (2009). Cold-induced silencing by long antisense transcripts of an *Arabidopsis* Polycomb target. Nature.

[76] Heo J.B., Sung S. (2011). Vernalization-mediated epigenetic silencing by a long intronic noncoding RNA. Science.

[77] Liu F., Marquardt S., Lister C., Swiezewski S., Dean C. (2010). Targeted 3' processing of antisense transcripts triggers *Arabidopsis* FLC chromatin silencing. Science.

[78] Ariel F., Jegu T., Latrasse D., Romero-Barrios N., Christ A., Benhamed M., Crespi M. (2014). Noncoding transcription by alternative RNA polymerases dynamically regulates an auxin-driven chromatin loop. Mol. Cell.

[79] Latrasse D., Germann S., Houba-Herin N., Dubois E., Bui-Prodhomme D., Hourcade D., Juul-Jensen T., le Roux C., Majira A., Simoncello N. (2011). Control of flowering and cell fate by LIF2, an RNA binding partner of the polycomb complex component LHP1. PLoS ONE.

[80] Davidovich C., Wang X., Cifuentes-Rojas C., Goodrich K.J., Gooding A.R., Lee J.T., Cech T.R. (2015). Toward a consensus on the binding specificity and promiscuity of PRC2 for RNA. Mol. Cell.

[81] Yuan W., Wu T., Fu H., Dai C., Wu H., Liu N., Li X., Xu M., Zhang Z., Niu T. (2012). Dense chromatin activates Polycomb repressive complex 2 to regulate H3 lysine 27 methylation. Science.

[82] Bracken A.P., Dietrich N., Pasini D., Hansen K.H., Helin K. (2006). Genome-wide mapping of Polycomb target genes unravels their roles in cell fate transitions. Genes Dev..

[83] Tolhuis B., de Wit E., Muijrers I., Teunissen H., Talhout W., van Steensel B., van Lohuizen M. (2006). Genome-wide profiling of PRC1 and PRC2 Polycomb chromatin binding in *Drosophila melanogaster*. Nat. Genet..

[84] Bouyer D., Roudier F., Heese M., Andersen E.D., Gey D., Nowack M.K., Goodrich J., Renou J.P., Grini P.E., Colot V. (2011). Polycomb repressive complex 2 controls the embryo-to-seedling phase transition. PLoS Genet..

[85] Oh S., Park S., van Nocker S. (2008). Genic and global functions for Paf1C in chromatin modification and gene expression in *Arabidopsis*. PLoS Genet..

[86] Zhang K., Sridhar V.V., Zhu J., Kapoor A., Zhu J.K. (2007). Distinctive core histone post-translational modification patterns in *Arabidopsis thaliana*. PLoS ONE.

[87] Lafos M., Kroll P., Hohenstatt M.L., Thorpe F.L., Clarenz O., Schubert D. (2011). Dynamic regulation of H3K27 trimethylation during *Arabidopsis* differentiation. PLoS Genet..

[88] Mohn F., Weber M., Rebhan M., Roloff T.C., Richter J., Stadler M.B., Bibel M., Schubeler D. (2008). Lineage-specific polycomb targets and *de novo* DNA methylation define restriction and potential of neuronal progenitors. Mol. Cell.

[89] Filion G.J., van Bemmel J.G., Braunschweig U., Talhout W., Kind J., Ward L.D., Brugman W., de Castro I.J., Kerkhoven R.M., Bussemaker H.J. (2010). Systematic protein location mapping reveals five principal chromatin types in *Drosophila* cells. Cell.

[90] Ernst J., Kheradpour P., Mikkelsen T.S., Shoresh N., Ward L.D., Epstein C.B., Zhang X., Wang L., Issner R., Coyne M. (2011). Mapping and analysis of chromatin state dynamics in nine human cell types. Nature.

[91] Sequeira-Mendes J., Araguez I., Peiro R., Mendez-Giraldez R., Zhang X., Jacobsen S.E., Bastolla U., Gutierrez C. (2014). The functional topography of the *Arabidopsis* genome is organized in a reduced number of linear motifs of chromatin states. Plant Cell.

[92] Roudier F., Ahmed I., Berard C., Sarazin A., Mary-Huard T., Cortijo S., Bouyer D., Caillieux E., Duvernois-Berthet E., Al-Shikhley L. (2011). Integrative epigenomic mapping defines four main chromatin states in *Arabidopsis*. EMBO J..

[93] Francis N.J., Saurin A.J., Shao Z., Kingston R.E. (2001). Reconstitution of a functional core polycomb repressive complex. Mol. Cell.

[94] Grau D.J., Chapman B.A., Garlick J.D., Borowsky M., Francis N.J., Kingston R.E. (2011). Compaction of chromatin by diverse Polycomb group proteins requires localized regions of high charge. Genes Dev..

[95] Eskeland R., Leeb M., Grimes G.R., Kress C., Boyle S., Sproul D., Gilbert N., Fan Y., Skoultchi A.I., Wutz A. (2010). Ring1B compacts chromatin structure and represses gene expression independent of histone ubiquitination. Mol. Cell.

[96] Shu H., Wildhaber T., Siretskiy A., Gruissem W., Hennig L. (2012). Distinct modes of DNA accessibility in plant chromatin. Nat. Commun..

[97] Bell O., Schwaiger M., Oakeley E.J., Lienert F., Beisel C., Stadler M.B., Schubeler D. (2010). Accessibility of the *Drosophila* genome discriminates PcG repression, H4K16 acetylation and replication timing. Nat. Struct. Mol. Biol..

[98] Canzio D., Chang E.Y., Shankar S., Kuchenbecker K.M., Simon M.D., Madhani H.D., Narlikar G.J., Al-Sady B. (2011). Chromodomain-mediated oligomerization of HP1 suggests a nucleosome-bridging mechanism for heterochromatin assembly. Mol. Cell.

[99] Canzio D., Liao M., Naber N., Pate E., Larson A., Wu S., Marina D.B., Garcia J.F., Madhani H.D., Cooke R. (2013). A conformational switch in HP1 releases auto-inhibition to drive heterochromatin assembly. Nature.

[100] Teif V.B., Kepper N., Yserentant K., Wedemann G., Rippe K. (2015). Affinity, stoichiometry and cooperativity of heterochromatin protein 1 (HP1) binding to nucleosomal arrays. J. Phys. Condens. Matter.

[101] Blein J.P., Coutos-Thevenot P., Marion D., Ponchet M. (2002). From elicitins to lipid-transfer proteins: A new insight in cell signalling involved in plant defence mechanisms. Trends Plant Sci..

[102] Cheutin T., Cavalli G. (2014). Polycomb silencing: From linear chromatin domains to 3D chromosome folding. Curr. Opin. Genet. Dev..

[103] De Wit E., de Laat W. (2012). A decade of 3C technologies: Insights into nuclear organization. Genes Dev..

[104] Hovel I., Louwers M., Stam M. (2012). 3C technologies in plants. Methods.

[105] Del Prete S., Arpon J., Sakai K., Andrey P., Gaudin V. (2014). Nuclear architecture and chromatin dynamics in interphase nuclei of *Arabidopsis thaliana*. Cytogenet. Genome Res..

[106] Lanzuolo C., Roure V., Dekker J., Bantignies F., Orlando V. (2007). Polycomb response elements mediate the formation of chromosome higher-order structures in the bithorax complex. Nat. Cell Biol..

[107] Tiwari V.K., McGarvey K.M., Licchesi J.D., Ohm J.E., Herman J.G., Schubeler D., Baylin S.B. (2008). PcG proteins, DNA methylation, and gene repression by chromatin looping. PLoS Biol..

[108] Williamson I., Berlivet S., Eskeland R., Boyle S., Illingworth R.S., Paquette D., Dostie J., Bickmore W.A. (2014). Spatial genome organization: Contrasting views from chromosome conformation capture and fluorescence *in situ* hybridization. Genes Dev..

[109] Comet I., Schuettengruber B., Sexton T., Cavalli G. (2011). A chromatin insulator driving three-dimensional Polycomb response element (PRE) contacts and Polycomb association with the chromatin fiber. Proc. Natl. Acad. Sci. USA.

[110] Li H.B., Ohno K., Gui H., Pirrotta V. (2013). Insulators target active genes to transcription factories and polycomb-repressed genes to Polycomb bodies. PLoS Genet..

[111] Zhu D., Rosa S., Dean C. (2014). Nuclear organization changes and the epigenetic silencing of FLC during vernalization. J. Mol. Biol..

[112] Louwers M., Bader R., Haring M., van Driel R., de Laat W., Stam M. (2009). Tissue- and expression level-specific chromatin looping at maize *b1* epialleles. Plant Cell.

[113] Crevillen P., Sonmez C., Wu Z., Dean C. (2013). A gene loop containing the floral repressor FLC is disrupted in the early phase of vernalization. EMBO J..

[114] Rosa S., de Lucia F., Mylne J.S., Zhu D., Ohmido N., Pendle A., Kato N., Shaw P., Dean C. (2013). Physical clustering of FLC alleles during Polycomb-mediated epigenetic silencing in vernalization. Genes Dev..

[115] Tolhuis B., Blom M., Kerkhoven R.M., Pagie L., Teunissen H., Nieuwland M., Simonis M., de Laat W., van Lohuizen M., van Steensel B. (2011). Interactions among Polycomb domains are guided by chromosome architecture. PLoS Genet..

[116] Simonis M., Klous P., Splinter E., Moshkin Y., Willemsen R., de Wit E., van Steensel B., de Laat W. (2006). Nuclear organization of active and inactive chromatin domains uncovered by chromosome conformation capture-on-chip (4C). Nat. Genet..

[117] Bello B., Holbro N., Reichert H. (2007). Polycomb group genes are required for neural stem cell survival in postembryonic neurogenesis of *Drosophila*. Development.

[118] Dixon J.R., Selvaraj S., Yue F., Kim A., Li Y., Shen Y., Hu M., Liu J.S., Ren B. (2012). Topological domains in mammalian genomes identified by analysis of chromatin interactions. Nature.

[119] Nora E.P., Lajoie B.R., Schulz E.G., Giorgetti L., Okamoto I., Servant N., Piolot T., van Berkum N.L., Meisig J., Sedat J. (2012). Spatial partitioning of the regulatory landscape of the X-inactivation centre. Nature.

[120] Sexton T., Yaffe E., Kenigsberg E., Bantignies F., Leblanc B., Hoichman M., Parrinello H., Tanay A., Cavalli G. (2012). Three-dimensional folding and functional organization principles of the *Drosophila* genome. Cell.

[121] Lieberman-Aiden E., van Berkum N.L., Williams L., Imakaev M., Ragoczy T., Telling A., Amit I., Lajoie B.R., Sabo P.J., Dorschner M.O. (2009). Comprehensive mapping of long-range interactions reveals folding principles of the human genome. Science.

[122] Ciabrelli F., Cavalli G. (2014). Chromatin-driven behavior of topologically associating domains. J. Mol. Biol..

[123] Bantignies F., Roure V., Comet I., Leblanc B., Schuettengruber B., Bonnet J., Tixier V., Mas A., Cavalli G. (2011). Polycomb-dependent regulatory contacts between distant Hox loci in *Drosophila*. Cell.

[124] Hou C., Li L., Qin Z.S., Corces V.G. (2012). Gene density, transcription, and insulators contribute to the partition of the *Drosophila* genome into physical domains. Mol. Cell.

[125] Lan X., Witt H., Katsumura K., Ye Z., Wang Q., Bresnick E.H., Farnham P.J., Jin V.X. (2012). Integration of Hi-C and ChIP-seq data reveals distinct types of chromatin linkages. Nucleic Acids Res..

[126] Noordermeer D., Leleu M., Splinter E., Rougemont J., de Laat W., Duboule D. (2011). The dynamic architecture of Hox gene clusters. Science.

[127] Feng S., Cokus S.J., Schubert V., Zhai J., Pellegrini M., Jacobsen S.E. (2014). Genome-wide Hi-C analyses in wild-type and mutants reveal high-resolution chromatin interactions in *Arabidopsis*. Mol. Cell.

[128] Wang C., Liu C., Roqueiro D., Grimm D., Schwab R., Becker C., Lanz C., Weigel D. (2015). Genome-wide analysis of local chromatin packing in *Arabidopsis thaliana*. Genome Res..

[129] Grob S., Schmid M.W., Grossniklaus U. (2014). Hi-C analysis in *Arabidopsis* identifies the KNOT, a structure with similarities to the flamenco locus of *Drosophila*. Mol. Cell.

[130] Moissiard G., Cokus S.J., Cary J., Feng S., Billi A.C., Stroud H., Husmann D., Zhan Y., Lajoie B.R., McCord R.P. (2012). MORC family ATPases required for heterochromatin condensation and gene silencing. Science.

[131] Grimaud C., Bantignies F., Pal-Bhadra M., Ghana P., Bhadra U., Cavalli G. (2006). RNAi components are required for nuclear clustering of Polycomb group response elements. Cell.

[132] Ren X., Vincenz C., Kerppola T.K. (2008). Changes in the distributions and dynamics of Polycomb repressive complexes during embryonic stem cell differentiation. Mol. Cell. Biol..

[133] Saurin A.J., Shiels C., Williamson J., Satijn D.P., Otte A.P., Sheer D., Freemont P.S. (1998). The human Polycomb group complex associates with pericentromeric heterochromatin to form a novel nuclear domain. J. Cell Biol..

[134] Vandenbunder B., Fourre N., Leray A., Mueller F., Volkel P., Angrand P.O., Heliot L. (2014). PRC1 components exhibit different binding kinetics in Polycomb bodies. Biol. Cell.

[135] Gonzalez I., Mateos-Langerak J., Thomas A., Cheutin T., Cavalli G. (2014). Identification of regulators of the three-dimensional Polycomb organization by a microscopy-based genome-wide RNAi screen. Mol. Cell.

[136] Gambetta M.C., Muller J. (2014). O-GlcNAcylation prevents aggregation of the Polycomb group repressor polyhomeotic. Dev. Cell.

[137] Libault M., Tessadori F., Germann S., Snijder B., Fransz P., Gaudin V. (2005). The *Arabidopsis* LHP1 protein is a component of euchromatin. Planta.

[138] Parry G. (2015). The plant nuclear envelope and regulation of gene expression. J. Exp. Bot..

[139] Tamura K., Goto C., Hara-Nishimura I. (2015). Recent advances in understanding plant nuclear envelope proteins involved in nuclear morphology. J. Exp. Bot..

[140] Zhou X., Groves N.R., Meier I. (2015). Plant nuclear shape is independently determined by the SUN-WIP-WIT2-myosin XI-i complex and CRWN1. Nucleus.

[141] Guelen L., Pagie L., Brasset E., Meuleman W., Faza M.B., Talhout W., Eussen B.H., de Klein A., Wessels L., de Laat W. (2008). Domain organization of human chromosomes revealed by mapping of nuclear lamina interactions. Nature.

[142] Pickersgill H., Kalverda B., de Wit E., Talhout W., Fornerod M., van Steensel B. (2006). Characterization of the *Drosophila melanogaster* genome at the nuclear lamina. Nat. Genet..

[143] Van Steensel B., Henikoff S. (2000). Identification of *in vivo* DNA targets of chromatin proteins using tethered dam methyltransferase. Nat. Biotechnol..

[144] Germann S., Gaudin V. (2011). Mapping *in vivo* protein-DNA interactions in plants by DamID, a DNA adenine methylation-based method. Methods Mol. Biol..

[145] Peric-Hupkes D., Meuleman W., Pagie L., Bruggeman S.W., Solovei I., Brugman W., Graf S., Flicek P., Kerkhoven R.M., van Lohuizen M. (2010). Molecular maps of the reorganization of genome-nuclear lamina interactions during differentiation. Mol. Cell.

[146] Ikegami K., Egelhofer T.A., Strome S., Lieb J.D. (2010). Caenorhabditis elegans chromosome arms are anchored to the nuclear membrane via discontinuous association with LEM-2. Genome Biol..

[147] Filion G.J., van Steensel B. (2010). Reassessing the abundance of H3K9me2 chromatin domains in embryonic stem cells. Nat. Genet..

[148] Shevelyov Y.Y., Lavrov S.A., Mikhaylova L.M., Nurminsky I.D., Kulathinal R.J., Egorova K.S., Rozovsky Y.M., Nurminsky D.I. (2009). The B-type lamin is required for somatic repression of testis-specific gene clusters. Proc. Natl. Acad. Sc.i USA.

[149] Towbin B.D., Meister P., Pike B.L., Gasser S.M. (2010). Repetitive transgenes in *C. elegans* accumulate heterochromatic marks and are sequestered at the nuclear envelope in a copy-number- and lamin-dependent manner. Cold Spring Harb. Symp. Quant. Biol..

[150] Andrulis E.D., Neiman A.M., Zappulla D.C., Sternglanz R. (1998). Perinuclear localization of chromatin facilitates transcriptional silencing. Nature.

[151] Dialynas G., Speese S., Budnik V., Geyer P.K., Wallrath L.L. (2010). The role of *Drosophila* Lamin C in muscle function and gene expression. Development.

[152] Finlan L.E., Sproul D., Thomson I., Boyle S., Kerr E., Perry P., Ylstra B., Chubb J.R., Bickmore W.A. (2008). Recruitment to the nuclear periphery can alter expression of genes in human cells. PLoS Genet..

[153] Reddy K.L., Zullo J.M., Bertolino E., Singh H. (2008). Transcriptional repression mediated by repositioning of genes to the nuclear lamina. Nature.

[154] Casolari J.M., Brown C.R., Drubin D.A., Rando O.J., Silver P.A. (2005). Developmentally induced changes in transcriptional program alter spatial organization across chromosomes. Genes Dev..

[155] Kundu S., Horn P.J., Peterson C.L. (2007). SWI/SNF is required for transcriptional memory at the yeast GAL gene cluster. Genes Dev..

[156] Schmid M., Arib G., Laemmli C., Nishikawa J., Durussel T., Laemmli U.K. (2006). Nup-PI: The nucleopore-promoter interaction of genes in yeast. Mol. Cell.

[157] Brickner J.H., Walter P. (2004). Gene recruitment of the activated INO1 locus to the nuclear membrane. PLoS Biol..

[158] Solovei I., Wang A.S., Thanisch K., Schmidt C.S., Krebs S., Zwerger M., Cohen T.V., Devys D., Foisner R., Peichl L. (2013). LBR and lamin A/C sequentially tether peripheral heterochromatin and inversely regulate differentiation. Cell.

[159] Wang J., Kumar R.M., Biggs V.J., Lee H., Chen Y., Kagey M.H., Young R.A., Abate-Shen C. (2011). The Msx1 homeoprotein recruits polycomb to the nuclear periphery during development. Dev. Cell.

[160] Fedorova E., Sadoni N., Dahlsveen I.K., Koch J., Kremmer E., Eick D., Paro R., Zink D. (2008). The nuclear organization of Polycomb/Trithorax group response elements in larval tissues of *Drosophila melanogaster*. Chromosome Res..

[161] Feng C.M., Qiu Y., van Buskirk E.K., Yang E.J., Chen M. (2014). Light-regulated gene repositioning in *Arabidopsis*. Nat. Commun..

[162] Jung J.H., Park C.M. (2013). HOS1-mediated activation of FLC via chromatin remodeling under cold stress. Plant Signal. Behav..

